# Vasodilator Activity of the Essential Oil from Aerial Parts of *Pectis brevipedunculata* and Its Main Constituent Citral in Rat Aorta

**DOI:** 10.3390/molecules18033072

**Published:** 2013-03-07

**Authors:** Sharlene Lopes Pereira, André Mesquita Marques, Roberto Takashi Sudo, Maria Auxiliadora Coelho Kaplan, Gisele Zapata-Sudo

**Affiliations:** 1 Drug Development Program, Biomedical Science Institute, Federal University of Rio de Janeiro, Carlos Chagas Filho Avenue 373, 21941-902, Rio de Janeiro, RJ, Brazil; E-Mails: sharlene.pereira@yahoo.com.br (S.P.); rtsudo@icb.ufrj.br (R.S.); 2 Natural Products Research Nucleus, Health Science Center, Federal University of Rio de Janeiro, Carlos Chagas Filho Avenue 373, 21941-902, Rio de Janeiro, RJ, Brazil; E-Mails: andrefarmaciarj@yahoo.com.br (A.M.); makaplan@uol.com.br (M.A.K.)

**Keywords:** *Pectis brevipedunculata*, citral, vasodilator effect, l-type Ca^2+^ channel, NO/cyclic GMP pathway

## Abstract

The essential oil of *Pectis brevipedunculata* (EOPB), a Brazilian ornamental aromatic grass, is characterized by its high content of citral (81.9%: neral 32.7% and geranial 49.2%), limonene (4.7%) and α-pinene (3.4%). Vasodilation induced by EOPB and isolated citral was investigated in pre-contracted vascular smooth muscle, using thoracic aorta from Wistar Kyoto (WKY) rats which was prepared for isometric tension recording. EOPB promoted intense relaxation of endothelium-intact and denuded aortic rings with the concentration to induce 50% of the maximal relaxation (IC_50_) of 0.044% ± 0.006% and 0.093% ± 0.015% (*p* < 0.05), respectively. The IC_50_ values for citral in endothelium-intact and denuded rings were 0.024% ± 0.004% and 0.021% ± 0.004%, respectively (*p* > 0.05). In endothelium-intact aorta, EOPB-induced vasorelaxation was significantly reduced by l-NAME, a nitric oxide synthase inhibitor. The vasodilator activity of citral was increased in the KCl-contracted aorta and citral attenuated the contracture elicited by Ca^2+^ in depolarized aorta. EOPB and citral elicited vasorelaxation on thoracic aorta by affecting the NO/cyclic GMP pathway and the calcium influx through voltage-dependent l-type Ca^2+^ channels, respectively.

## 1. Introduction

In Brazil, many plant species are known as lemongrass due to the citric fragrance from their volatile constituents. *Pectis* is a genus composed by small herb plants of the daisy family generally considered as weeds in current usage [1]. Some species of this genus have this citrus-like scent due to the presence of citral and limonene in their volatile composition. Species of *Pectis* are native to the Americas, including the Caribbean, being found also in the Pacific Islands [2]. These species have been found in a variety of hot dry habitats, including deserts, tropical and subtropical grasslands and tropical beaches [3]. Due to their citrus-like smell, herb infusions of *Pectis elongata* have been popularly utilized in tea and as spices in French Guyana to replace the lemongrass species, *Cymbopogon citratus*, Poaceae [4]. In southeast and northeast regions of Brazil, some *Pectis* species have traditional use as infusions or juice drink preparations for hypertension, stomach disorders and colds [5]. Calmative and analgesic properties were also reported for some *Pectis* tea preparations [6,7].

*Pectis brevipedunculata*, a Brazilian ornamental aromatic grass, is one of the “lemongrass odor” correlated species. Its essential oil content is characterized by the high percentage of citral (up to 78%). This monoterpene aldehyde fraction is normally composed by a mixture of the two geometric *cis*- and *trans*-isomers: geranial and neral. Many biological activities were described to this aldehyde monoterpene fraction. Bacteriostatic and fungistatic properties have already been related to essential oils rich in citral [8,9,10,11]. In rats, cardiovascular effects as transient hypotension and bradycardia were induced by the citral-rich essential oil obtained from lemon grass, *Cymbopogon citratus* [12]. In patients with essential hypertension, aromatherapy with a mixture of essential oils rich in limonene and citral was effective in lowering systolic blood pressure and sympathetic nerve system activity [13]. Recently, Devi and co-workers described that methanolic extract of *Cymbopogon citratus* and citral induced vasodilation in isolated thoracic rat aorta and demonstrated that citral may affect the intracellular calcium concentration on vascular smooth muscle cells [14]. The present work investigated the potential vascular effects of the essential oil of *Pectis brevipedunculata* (EOPB) and its major constituent citral on rat isolated thoracic aorta and elucidated the mechanism underlying this activity.

## 2. Results and Discussion

### 2.1. Chemistry

**Figure 1:**
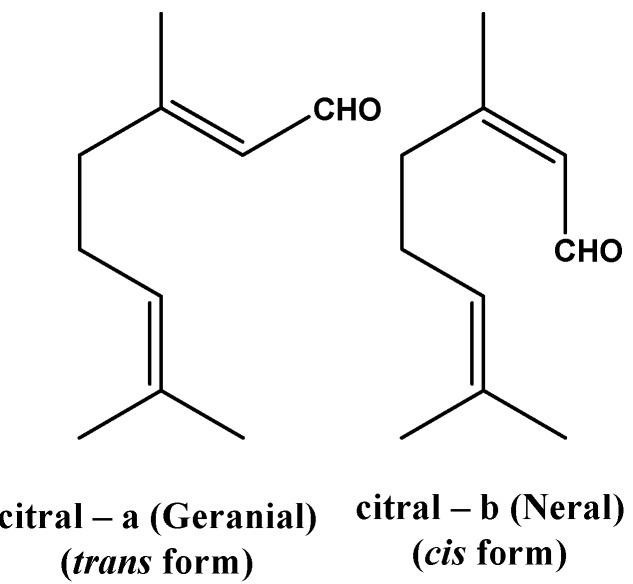
Structure of citral geometric isomers.

The relative amount (%v/w) of the essential oil (EO) of fresh aerial parts of *P. brevipedunculata* was 0.4% and the chemical analysis of the investigated EO is presented in Table 1. The chemical composition of *P. brevipedunculata* volatile fractions consists of monoterpene compounds, hydrocarbons, sesquiterpenes, alcohols and aldehydes. The GC-FID and CG/MS methods were used to identify and quantify these compounds. The essential oil was characterized by a high percentage of citral (81.7%: neral 32.5% and geranial 49.2%; Figure 1), followed by limonene (4.5%) and α-pinene (3.3%). The high percentage of citral in the EO of *P. brevipedunculata* ranges from 80% to 90% year-round placing this native Brazilian species as one potencial natural source of citral rich essential oil. Alcohol *cis* and *trans* enantiomers derivatives were also detected: nerol and geraniol, corresponding to 1.5% and 5.1% in the whole oil, respectively. Citral is the major component (>80.0%) of *P. brevipedunculata* EO occurring in lemon grass (*C. citratus*) where citral is found up to 70.0% in the mixture. In addition, the presence of limonene in the EO of *P. brevipedunculata* contributes to enhance the lemon fragrance of the composition.

**Table 1:** Identified Compounds in the aerial parts essential oil of *P. brevipedunculata*.

Compounds	^a^ RI^ Lit^	^b^ RI	FRESH HD %	Identification
α-Pinene	939	938	3.3	RI, GCMS
Limonene	1029	1032	4.5	RI, GCMS
Longipenene epoxide	1089	1186	1.4	RI, GCMS
Nerol	1233	1233	1.5	RI, GCMS
**Neral**	1247	1248	**32.5**	RI, GCMS
Geraniol	1276	1260	5.1	RI, GCMS
**Geranial**	1277	1278	**49.2**	RI, GCMS
1-Tridecene	1293	1292	0.8	RI, GCMS
β-Elemene	1393	1389	0.7	RI, GCMS
β-Farnesene	1445	1418	0.2	RI, GCMS
α-Cariophylene	1457	1455	0.3	RI, GCMS
Sum of identified peaks			99.5%	

^a^ RI^ Lit^: Literature Retention Indices [15] ^b^ RI: Experimental Retention Indices; HD: Hydrodistillation.

### 2.2. Pharmacology

#### 2.2.1. Effects of EOPB and Citral on Vascular Smooth Muscle

**Figure 2:**
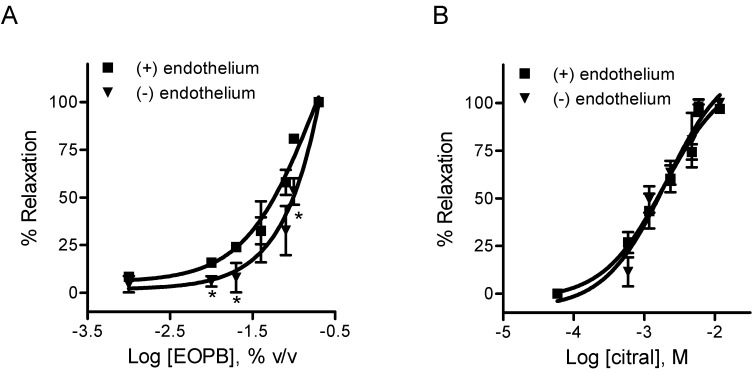
Concentration-response curves for essential oil of *Pectis brevipedunculata* (EOPB) (**a**) and citral (**b**) in endothelium-intact and denuded aorta from Wistar-Kyoto rats, precontracted with phenylephrine. Data are mean ± SEM (n = 4). * *p* < 0.05 *vs.* (+) endothelium. (+): intact endothelium; (−): denuded endothelium.

The vasodilator activity of EOPB or citral was investigated in aortic rings from WKY rats. Both EOPB and citral induced relaxation of the phenylephrine-precontracted aorta in a concentration-dependent manner (Figure 2). The concentration of EOPB to induce 50% of the maximal relaxation (IC_50_) in the phenylephrine-induced contraction of endothelium-intact and denuded rings from WKY rats were 0.044 ± 0.006% (n = 4) and 0.093 ± 0.015%, respectively (n = 4, *p* < 0.05, Table 2). However, the IC_50_ values for citral in endothelium-intact and -denuded rings were 1.42 ± 0.26 mM (n = 4) and 1.33 ± 0.18 mM, respectively (n = 4, *p* > 0.05, Table 2).

**Table 2:** Concentrations of essential oil of *Pectis brevipedunculata* (EOPB) or citral to induce 50% of the maximal relaxation (IC_50_) in the phenylephrine-induced contraction in aortic rings from Wistar Kyoto rats.

	IC_50_	
	EOPB (%)	Citral (mM)
With endothelium	0.044 ± 0.006	1.42 ± 0.26
Without endothelium	0.093 ± 0.015 ^a^	1.33 ± 0.18

^a^ *p* < 0.05 compared to EOPB with intact aorta; Data are expressed as the mean ± S.E.M., n = 5

**Figure 3:**
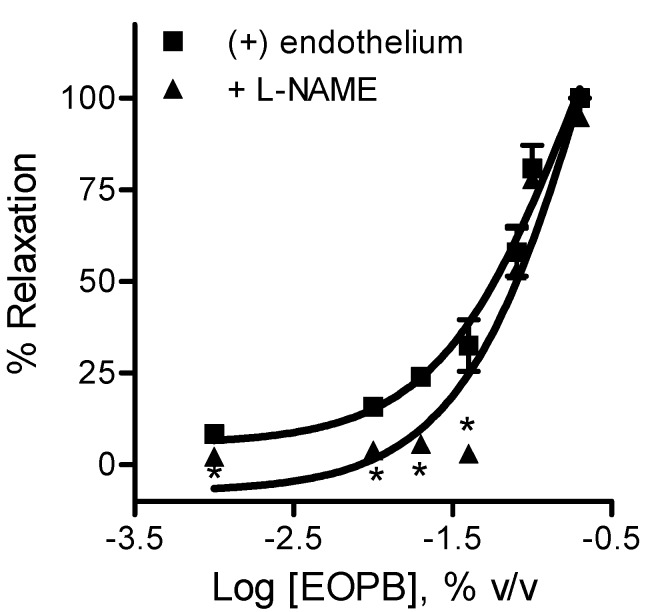
Effects of essential oil of *Pectis brevipedunculata* (EOPB) on endothelium-intact aortic rings from WKY rats in the presence of l-NAME (100 μM). Concentration-response curves for EPPB in aorta precontracted with phenylephrine. Data are mean ± SEM (n = 4). * *p* < 0.05 *vs.* (+) endothelium.

Considering the endothelial involvement in the relaxation of EOPB in aortic rings, we investigated the possible involvement of the NO/cyclic GMP pathway in the endothelium-dependent mechanism of EOPB. The incubation of aortic rings with l-NAME (Figure 3) significantly reduced the IC_50_ for EOPB from 0.044 ± 0.006% (n = 4) to 0.081 ± 0.013% (n = 4, *p* < 0.05). 

**Figure 4:**
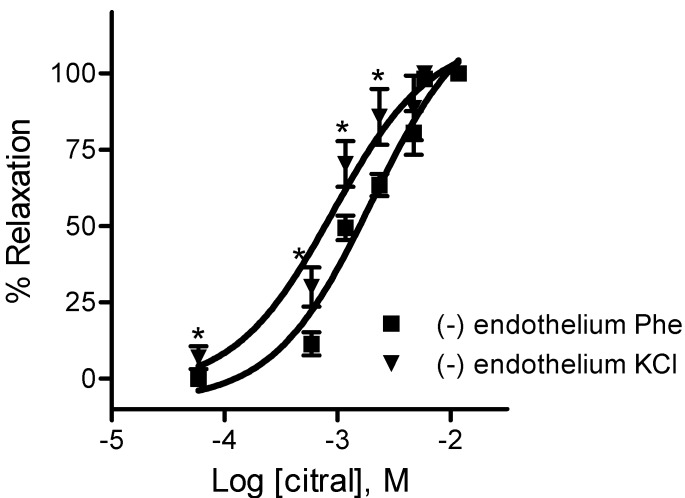
Concentration-response curves for citral in endothelium-denuded aorta from WKY rats, precontracted with 10 µM phenylephrine (Phe) or 80 mM KCl. Data are mean ± SEM (n = 4). * *p* < 0.05 *vs.* (−) endothelium Phe.

In another set of experiments, the effect of citral on the K^+^-induced contraction was investigated. When endothelium-denuded aortic rings were contracted with high K^+^ extracellular concentration (80 mM), the relaxation after exposure to citral was increased (Figure 4). Under these conditions, the concentration-response curve was shifted leftwards when compared to phenylephrine-contracted aortas. The IC_50_ value for citral was 0.69 ± 0.08 mM in KCl-contracted aortic rings (n = 4, *p* < 0.05 *vs.* phenylephrine-contracted aorta).

**Figure 5:**
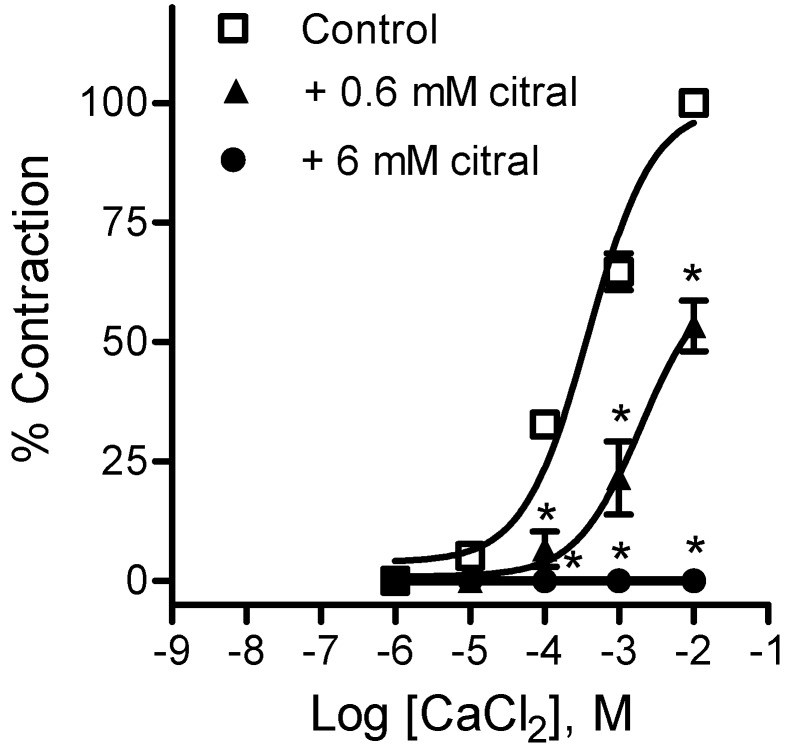
Effects of citral on Ca^2+^-induced contraction of depolarized endothelium-denuded aorta from WKY rats. Contractions were taken as 100% in the presence of 10^−2^ M CaCl_2_ (control curve). Data are mean ± SEM (n = 4). * *p* < 0.01 *vs.* control.

Based on the fact that the citral potency was increased when aortic rings were contracted by membrane depolarization, we investigated whether this compound impaired extracellular Ca^2+^ influx through voltage-dependent Ca^2+^ channels. Concentration-response curves for Ca^2+^ were obtained in the absence and presence of citral, in depolarized aorta from WKY rats. Citral effectively attenuated the contractile response to Ca^2+^ in aortic rings. Maximal contraction induced by 10 mM CaCl_2_ was reduced to 53.38 ± 5.33% of the control (n = 4, *p* < 0.01) or completely abolished when in the presence of 0.6 mM and 6 mM of citral (Figure 5).

In Brazil, *Cymbopogon citratus* (lemongrass) is used in the folk medicine as a high blood pressure treatment. Many hypertensive patients drink *Cymbopogon citratus* tea daily due to its calmative, anxyolitic as well as anti-hypertensive properties. The citrus scent of *C. citratus* essential oil is mainly a results of the high content of neral and geranial (citral >70.0%) in its composition, followed by geraniol. The main components of the volatile fractions in the *Pectis brevipedunculata* EO are neral and geranial (citral > 80.0%) followed by nerol and geraniol as minor derivatives. The EO of *P. brevipedunculata* contains 80%–90% of citral, placing this native Brazilian plant as a potential natural source of citral-rich essential oil. Similar fragrances common to *Pectis* species could explain the traditional use of that lemongrass. In agreement with this similarity, ethnopharmacological studies have reported the traditional use of infusions of *P. brevipedunculata* and *P. jangadensis* by some communities in Brazil to produce anxyolisis [7]. In the northeast of the country, *P. elongata* Kunt and *P. linifolia* L. var. *linifolia* have been used in the folk medicine to treat hypertension and gastric diseases, while *P. oligocephala* (Gardner) Sch.Bip. is usually indicated to treat cold [4,5]. The qualitative and quantitative analysis of the major compounds in the EO of lemon grass and *P. brevipedunculata* are quite similar, showing citral as main compound in the mixture (citral > 70.0%) in both species. This fact could explain their use in folk medicine for the same purposes. Unfortunately, no data is available in literature concerning the phytochemical and pharmacological profile of tea drinks extracted from *Pectis* species. However, some ethnopharmacological data are available for citral and tea preparations from *C. citratus.* Despite the strong popular indication, some discrepant results can be found. Caluscusin has found that twice-a-day intake of lemongrass decoction had a significant effect on the mean arterial pressure, probably due to a diuretic action of the tea [16]. On the other hand, negative results were observed in rodents [17,18,19] and in humans [20]. That controversy could be explained by different chemotypes of lemongrass evaluated, since there are at least two varieties: East Indian (roughly equal amounts of myrcene and citral) and the West Indian type (little myrcene but high amounts of citral). Nevertheless, all studies suggest that lemongrass infusions used in Brazilian folk medicine has no toxic properties [21].

Additionally, it should be considered that the effective dose of EO is still inconsistent with the amount of fresh leaves frequently used to make a cup of tea (2 to 10 g). Considering the average extraction yield of 0.5%, roughly 200 g of fresh leaves should be required for EO doses of 1.0 g/kg. Moreover, differences have been found in dosage regimen, typically in repeated or chronic intake of tea in comparison to acute treatment tested experimentally. In addition, different proportions of the EO could be found in tea preparation or infusions. Several secondary metabolites were already found in aqueous solutions of *C. citratus* such as saponins, sesquiterpenes, lactones, alcacaloids, tanins, steroids, triterpenes and flavonoids [22,23].

Shimono and co-workers reported that inhalation of a synthetic citral formulation reduced blood pressure. The antihypertensive agent comprises citral and linalol and has a mass ratio of citral to linalol of not less than 10:1 and not greater than 40:1. This formulation could be used as antihypertensive agent for medical drugs, foods, beverages and feeds. The mass ratio of 20:1 is preferred because the greatest effect was exhibited [24].

Although citral is reported as an antihypertensive agent, it is suggested that the concentration found in the small quantity of plant material used in a cup of tea could not be sufficient to the antihypertensive activity. Therefore, the reduction of blood pressure in humans observed in some studies may be related to the presence of other compounds in lemongrass tea.

Transient hypotension and bradycardia were previously reported in rats treated with the citral-rich essential oil obtained from *Cymbopogon citratus* [12]. Additionally, aromatherapy with a mixture of essential oils rich in limonene and citral was effective in lowering systolic blood pressure and sympathetic nerve system activity in hypertensive patients [13]. Recent studies regarding the vascular effects of methanolic extracts of *Cymbopogon citratus* and citral on isolated thoracic rat aorta suggested an impairment of the phenylephrine-induced contraction in Ca^2+^-free solution and a reduction of the CaCl_2_-induced contraction in endothelial intact and denuded aortic rings pretreated with citral [14]. These findings indicate an endothelium-independent vasorelaxation of citral and suggest that it may affect the intracellular calcium concentration by blocking the Ca^2+^ influx from the extracellular space possibly via receptor-operated calcium channels or the Ca^2+^ release from intracellular storage sites.

In the present study, we demonstrated an endothelium-independent vasodilator action elicited by citral on thoracic aorta from WKY rats. Citral concentration-dependently inhibited high K^+^ and phenylephrine-induced contractions. Moreover, the vasodilator effect of citral on KCl-induced contraction was more potent than on phenylephrine-induced contraction. It is well known that high K^+^-induced contraction in vascular smooth muscle is mediated by cell membrane depolarization and consequently an increase in Ca^2+^ influx through voltage-dependent l-type Ca^2+^ channels [25,26]. In contrast, phenylephrine-induced contraction is due to (1) Ca^2+^ influx through l-type Ca^2+^ channels, (2) Ca^2+^ influx through non-l-type Ca^2+^ channels such as receptor-operated calcium channels (ROC) [27], (3) enhancement of Ca^2+^ sensitivity [28], and (4) intracellular Ca^2+^ release from sarcoplasmic reticulum (RS) [29]. Phenylephrine activates α_1_-adrenergic receptors on vascular smooth muscle, leading to the formation of second messengers, inositol triphosphate (IP_3_) and diacylglycerol (DAG). IP_3_ promotes Ca^2+^ release from the RS and DAG activates the protein kinase C (PKC) which, in turn, phosphorylates and (1) activates ROC channels and promotes calcium influx; (2) inhibits K^+^ channels and leads to cellular depolarization which opens l-type calcium channels; (3) activates Raf–MEK–MAPK and RhoA/Rho-kinase pathways, enhancing the myofilament force sensitivity to Ca^2+^. Thus an l-type Ca^2+^ channel inhibitor induces less potent inhibition on phenylephrine-induced contraction than on depolarization-induced contraction. Secondly, citral impaired extracellular Ca^2+^ influx through voltage-dependent Ca^2+^ channels by reducing the contraction mediated by CaCl_2_ in depolarized vascular smooth muscle. Taken together, these results suggest that citral inhibits the extracellular Ca^2+^ influx through the blockade of voltage-dependent l-type Ca^2+^ channels.

In addition to the mechanism of citral described in the present work, which is related to the inhibition of l-type Ca^2+^ channels, the reduction of intracellular calcium concentration via receptor-operated calcium channels or the Ca^2+^ release from RS has already been described [14]. Therefore, further studies are required to establish whether citral interfere with intracellular excitation-contraction coupling of vascular smooth muscle, such as the myofilaments force sensitivity to Ca^2+^. Citral could decrease the myofilament sensitivity by activating the myosin light chain (MLC) phosphatase, which causes MLC dephosphorylation and reduces actin-myosin interaction. Alternatively, citral could inhibit the MLC kinase with consequent reduction of phosphorylated MLC and actin–myosin interaction. And also, could interfere with the formation of Ca^2+^-calmodulin complex and thus with the vascular smooth muscle contraction. Finally, citral could inhibit Raf-MEK-MAPK or RhoA/Rho-kinase pathways, decreasing myofilament force sensitivity and leading to vasorelaxation [25].

We also investigated the vascular effects of the essential oil of *Pectis brevipedunculata* (EOPB). This essential oil also induced a concentration-dependent vasodilation on thoracic aortic rings from WKY rats. Endothelium removal and the pretreatment with l-NAME caused partial inhibition of the EOPB vasorelaxant response. Nitric oxide is likely to be the endothelium-dependent component, since inhibition of the EOPB response by l-NAME was comparable to that by endothelium removal. Our findings suggest that EOPB-mediated vasorelaxation contained an endothelium-dependent component, involving the NO/cyclic GMP pathway. Additionally, in the presence of l-NAME, higher concentrations of EOPB which contains high concentration of citral, induced the same relaxation of the control indicating that the endothelium-independent component of EOPB-induced vasodilation could be related to the l-type calcium channels blockage. At high concentrations of EOPB, the endothelium-independent vasorelaxation should predominate in relation to the endothelium-dependent pathway.

Hayes and Markovic demonstrated that citral has antimicrobial activity against a range of Gram positive and Gram negative bacteria, but cytotoxicity assays suggest that citral is toxic to human liver- derived cells, skin cells and skin fibroblasts. Despite the described cytotoxic activity of citral, a product containing 1% lemon myrtle oil (rich in citral) was significantly less toxic to human skin cells and fibroblasts [30]. On the other hand, Dijoux and collaborators described the lack of phototoxic and cytotoxic effects of lemongrass essential oil (rich in citral) on murine fibroblast cells and rabbit cornea derived cells. Phototoxicity is a skin reaction caused by concurrent topical or systemic exposure to specific molecule and ultraviolet radiation. This study suggested a non-cytotoxic action of the main constituent of the lemongrass essential oil (citral) [31]. Additionally, Santoro *et al*. analyzed the anti-proliferative effect of lemongrass essential oil on three evolutive forms of *Trypanosoma cruzi*, which was effective against *T. cruzi* trypomastigotes and amastigotes. In this study, no cytotoxic effects were observed when mouse peritoneal macrophages were incubated with lemongrass essential oil at concentrations corresponding to the IC_50_ for trypomastigotes, suggesting a lack of cytotoxic effects against mammalian cell type [32]. Finally, according to Mesa-Arango *et al*., citral was cytotoxic on tumoral cells (human cervix epithelioid carcinoma cells) but not on non-tumoral cells (african green monkey kidney cells) [33]. Considering that some reports show the lack of cytotoxic effects of citral or essential oils, additional cytotoxicity experiments on different cell lines are needed to support these data. In our study, the vasorelaxant effect of citral and essential oil (EOPB) was not due to myocyte injury, since we observed the same contractile capacity of the aortic rings after exposing them to increasing concentrations of both citral and EOPB. This observation indicates the tissue integrity and a non-cytotoxic effect of citral in vascular smooth muscle cells.

## 3. Experimental

### 3.1. Chemistry

#### 3.1.1. Plant Material and Essential Oil Extraction

Aerial parts (100 g) of *Pectis bervipedunculata* were collected in Rio de Janeiro, RJ in April 2011. The botanical vouchers were identified by Dr. Roberto L. Esteves and kept at the Herbarium (HB) of the Rio de Janeiro National Museum under number 1007 (R). The fresh aerial parts were submitted to hydrodistillation for 2 h in a modified Clevenger-type apparatus. The obtained essential oil (EO) was dried over anhydrous sodium sulphate, yielding 0.4% w/w which was immediately stored in closed dark vials at 4 °C until analysis.

#### 3.1.2. GC-FID Analysis

Quantitative and qualitative analysis were carried out on a Shimadzu GC 2010 machine equipped with a ZB-1MS fused silica capillary column (30 m × 0.25 mm × 0.25 μm film thickness). The operating temperatures used were: injector 260 °C, detector 290 °C and column oven 60 °C up to 290 °C (10 °C/min). Hydrogen at 1.0 mL·min^−1^ was used as carrier gas. The percentages of the compounds were obtained by GC-FID analysis.

#### 3.1.3. GC-MS Analysis

Qualitative analyses were carried out on a GC-QP2010 PLUS Shimadzu machine with a ZB-5MS fused silica capillary column (30 m × 0.25 mm × 0.25 μm film thickness). The operating temperatures used were: injector 270 °C, detector 290 °C and column oven 60 °C up to 290 °C (3 °C/min). Helium at 1.0 mL·min^−1^ was used as carrier gas for GC/MS. The essential oil components were identified by comparison of their retention indices and mass spectra with published data [15] and computer matching with WILEY 275 and National Institute of Standards and Technology (NIST 3.0) libraries provided with the computer controlling the GC-MS system. The retention indices were calculated for all volatile constituents using the retention data of linear C8–C24 *n*-alkanes.

#### 3.1.4. Countercurrent Chromatography Separation Procedure

##### 3.1.4.1. Preparation of Two-Phase Solvent System and Sample Solutions

For HSCCC, we selected a two-phase solvent system composed of hexane/ACN (1:1, v/v). The distribution of the components in the EO was estimated by thin-layer chromatography (TLC, silica gel 60 F254 nm) with hexane/EtOAc (3:2) as the eluting solvent. The separation of compounds was observed under a UV lamp at 254nm and by spraying sulfuric acid/methanol reagent (1:1, v/v), followed by heating to assist visual estimation of the relative distribution of the compounds in each phase. The solvent mixture was hand mixed and thoroughly equilibrated in a separation funnel at the same temperature as in the vessel of HSCCC and the two phases were separated shortly before use. The sample solutions were prepared by diluting the essential oil in the mixture solution of lower and upper phases (1:1, v/v) of the used solvent system for HSCCC separation and vigorously hand mixing before the TLC analysis.

##### 3.1.4.2. Apparatus and Citral Separation Procedure

A CCC (model HSCCC No. 403, PC Inc., Potomac, MD, USA), consisting of a PTFE 80-mL coil, a HPLC pump (model M-45, Waters, Milford, MA, USA), a low-pressure injection valve (Rheodyne 5020, Cotati, CA, USA), and a PTFE 5-mL sample loop were used. This system was coupled to a fraction collector (model L-7650, Merck, Darmstadt, Germany) programmed to collect at 1-min intervals. Appropriate volumes of solvents hexane/ACN (1:1, v/v) were vigorously hand-mixed in a separatory funnel, transferred to a flask, degassed (ultrasonic bath) for 30 min. Isocratic elution was conducted in a tail-to-head manner, with the acetonitrile (lower phase) as stationary phase (normal elution mode). The coil was entirely filled with the lower phase of the solvent system with no rotation. Rotation of the coil was then started at 860 rpm, and the upper organic phase was pumped at a flow rate of 1.0 mL/min. Hydrodynamic equilibrium was attained prior to sample injection. The stationary phase retention was about 72.5%. The crude essential oil (1 mL) was dissolved in the biphasic solvent system and injected into to the apparatus separately. Fractions (240) of 1 ml each were collected in 200 min. The collected fractions were analyzed by TLC, GC – FID, GC-MS. About 400 μL of citral was obtained in high level of purity >98.0%.

### 3.2. Pharmacology

The protocols used in the present study were approved by Animal Care and Use Committee at Universidade Federal do Rio de Janeiro.

#### 3.2.1. Preparation of Aortic Rings and Protocols

Thoracic aortas were removed from 15- to 17-week-old male Wistar Kyoto rats (WKY), cleaned of connective tissue and prepared for isometric tension recording, as previously described [34]. Aortic rings of 3–4 mm in length were placed in chambers filled with physiological solution composed in mM of NaCl 123, KCl 4.7, MgCl_2_ 1.2, KH_2_PO_4_ 1.2, glucose 11.5, NaHCO_3_ 15.5, CaCl_2_ 1.2; bubbled with 95% O_2_/5% CO_2_ and maintained at 37 °C. After a 2 h equilibrium period of 1 g resting tension, the aortic rings were contracted with phenylephrine (10 µM), followed by exposure to acetylcholine (10 µM) to test the integrity of the endothelium. Acetylcholine-induced relaxation more than 80% denoted the presence of intact endothelium. In some experiments, EOPB or citral was tested in aortas in which the endothelium had been mechanically removed [34]. To investigate the ability of EOPB or citral to induce the relaxation of aortic rings from WKY rats, intact or endothelial-denuded rings were precontracted with a single concentration of phenylephrine (10 µM) and exposed to increasing concentrations of EOPB or citral. The vascular effects of citral were also evaluated in KCl-contracted denuded aortas (80 mM). The high K^+^ solution (80 mM KCl) was made by substituting NaCl with equimolar KCl.

To investigate the effects of citral on Ca^2+^ influx in the smooth muscle, denuded rings were equilibrated in Ca^2+^-free saline solution for 15 min. The solution then was replaced with Ca^2+^-free/high-K^+^ solution (80 mM). Aortic rings were kept in this depolarized state for 15 min, in the absence or presence of 0.6 or 6 mM citral, after which increasing concentrations of CaCl_2_ (10^−6^–10^−2^ M) were added.

In other experiments, endothelium-intact aortic rings from WKY rats were incubated for 20 min with 100 μM N^ω^-nitro-L-arginine methyl ester (l-NAME) [35], a selective nitric oxide synthase inhibitor, to investigate the possible mechanism involved in EOPB action. 

#### 3.2.2. Compounds

EOPB and citral (geranial and neral) were dissolved in distilled water. Phenylephrine, acetylcholine and N^ω^-nitro-L-arginine methyl ester (l-NAME) were purchased from Sigma Chemical Co. (St. Louis, MO, USA). All compounds were dissolved in distilled water. 

### 3.3. Statistical Analysis

All data are expressed as the mean ± standard error of the mean (SEM). The concentration necessary to reduce the phenylephrine-induced contraction by 50% (IC_50_) was determined for each experiment. The concentration-response curve was fitted to the following equation: y_max_ = y_min_ + a/(1 + e^−x−x^^0/b^), where y is the percentage of isometric tension; a = y_max_ − y_min_; b = slope; x_0_ = IC_50_. The variable x is the concentration of EOPB or citral to produce relaxation. Differences between 2 groups were determined with the unpaired Student’s *t-*test and were considered significant when *p* was <0.05.

## 4. Conclusions

In this work, we have demonstrated that *Pectis brevipedunculata* is a potential natural source of citral because it is the main component of its essential oil. The crude essential oil (EOPB) elicited vasorelaxation of thoracic aorta by affecting the NO/cyclic GMP pathway. In addition, our results suggest that citral reduced the calcium influx by the blockade of voltage-dependent l-type Ca^2+^ channels. Thus *Pectis brevipedunculata*, a Brazilian native plant, could be used to reduce high blood pressure.
